# Supplementary material for: Withania somnifera (L.) Dunal whole-plant extracts exhibited anti-sporotrichotic effects by destabilizing peripheral integrity of Sporothrix globosa yeast cells
Source: PLoS Negl Trop Dis. 2022 Jun 17;16(6):e0010484. doi: 10.1371/journal.pntd.0010484 (PMC9205525; doi:10.1371/journal.pntd.0010484)
Supplement: Attachment — Submitted filename: Itemized Response to Review Comments-Final.docx [file pntd.0010484.s002.docx]

**Itemized response to the review comments for the manuscript titled “Anti-Sporotrichotic Effects of *Withania somnifera* (L.) Dunal Whole-plant Extracts due to Peripheral Integrity Destabilization of *Sporothrix globosa* Yeast Cells”**

**(ID: PNTD-D-22-00025)**

Reviewer's Responses to Questions

Key Review Criteria Required for Acceptance

Methods
-Are the objectives of the study clearly articulated with a clear testable hypothesis stated?
-Is the study design appropriate to address the stated objectives?
-Is the population clearly described and appropriate for the hypothesis being tested?
-Is the sample size sufficient to ensure adequate power to address the hypothesis being tested?
-Were correct statistical analysis used to support conclusions?
-Are there concerns about ethical or regulatory requirements being met?

Reviewer #1: 1. The objectives of the study was clearly articulated with a clear testable hypothesis.
2. The study design was appropriate.
3. As in vitro study so no need of population definition.
4. Statistical analysis was supportive.

**Authors’ Response:** We take this opportunity to thank the reviewer for judging our manuscript in a favourable light, and indeed feel encouraged by the recognition of our efforts shown by the esteemed reviewer.

Reviewer #2: Include studies on the antisporotrichosis effects of other plants.
Review the study "Ramírez-Soto MC, Aguilar-Ancori EG, Tirado-Sánchez A, Bonifaz A. Ecological Determinants of Sporotrichosis Etiological Agents. J Fungi (Basel). 2018;4(3):95."
The objectives of the study are clearly articulated with a clear testable hypothesis stated.
The study design is appropriate to address the objectives.
There was not statistical analysis. What statistical tests did you use for comparisons?

**Authors’ Response:** We thank the reviewer for critically assessing our manuscript. As per the reviewer’s suggestion, we have now cited the study by Ramírez-Soto et al, 2018 in the revised manuscript as “Most medically relevant *Sporothrix* *spp* grow in soil (Ramírez-Soto et al., 2018).”. Kindly, refer to lines 78- 79 on page 4 of the revised manuscript. This suggestion by the reviewer has indeed helped in improving the body of evidence used to discuss our study. We are grateful for the reviewer’s appreciation of our study design and its experimental execution. We sincerely apologize for the inadvertent confusion regarding the statistical analyses done in this manuscript. However, we would like to politely mention here that we already dedicated a sub-section ‘Statistical analysis’ under the section ‘Methods’ in the original manuscript submitted which reads “Data represented as mean ± standard error of mean (SEM) using GraphPad Prism version 7.0 (GraphPad Prism 7.0, San Diego, CA, USA). Statistical significance of the observations was determined using in-built analysis option of the software. Observation was considered to be significant at p < 0.05.” Kindly, refer to lines 202 to 205 on page 10 of the of the original manuscript and lines 277 to 281 on page 13 of the revised one. Besides, types of statistical analyses conducted is individually mentioned the corresponding legends of the figures depicting data graphically.

**References:**

Ramírez-Soto, M. C., Aguilar-AWncori, E. G., Tirado-Sánchez, A., and Bonifaz, A. (2018). Ecological determinants of sporotrichosis etiological agents. J. Fungi 4, 1–11. doi:10.3390/jof4030095.

Reviewer #3: (No Response)

Reviewer #4: Questions in Methods sections: 1. Is fungal culture medium the same than BHI? or are you referring to a different medium? please specify and comment the composition of the medium. 2) The rationale behind ergosterol and sorbitol assays is not clear. Please specify. 3) I think the terms MIC50 and MIC80 are wrongly used here. 4) what were the standards used for the HPLC analysis and how is that you choose them? This section is not clear. how you identified withaferin, withanoline etc?
**Authors’ Response:** We thank the reviewer for raising the above queries in the method section and realized that responding these comments eventually infused more clarity into the methodologies followed in this study. We have provided a point-wise response below to the reviewer comments on method section:

1. *Sporothrix globosa* was cultured in Brain Heart Infusion (BHI) agar or broth, as per the requirement, in the entire study. This information is already provided under the sub-section ‘Chemicals, fungal media, and fungal strain’ of the ‘Method’ section of the original manuscript as “Fungal cells were reactivated from glycerol stock, first by streaking on brain heart infusion (BHI) agar plate for single colony and subsequently, by overnight culturing in BHI broth at 37 °C.” and has been retained unmodified in the revised manuscript except for addition of “All *S. globosa* cultures were either grown on BHI agar plates or in BHI broth.” at the end of the segment. Kindly refer to lines 154-156 on page 7 of the revised manuscript. Besides, for better clarity the culture media has been again mentioned under the subsection ‘Determination of Inhibitory Concentration through Broth Microdilution method’ of the ‘Method’ section in the revised manuscript, in line 181 on page 9.
2. The sorbitol protection and ergosterol binding assays are well-known methods of evaluating whether a cell permeability affecting antifungal agent targets the cell wall or the cell membrane (Mani-López et al., 2021). Sorbitol protection assay relies on the isotonic protection provided by 0.8 M sorbitol in the medium to the cells whose cell walls have been damaged by the test anti-fungal agent. Due to this protection, in the presence of sorbitol, the efficacy of the anti-fungal agent is apparently reduced as evident from increased IC_50_ value in comparison to that obtained for the same fungus in the absence of sorbitol. This clarification is now included in the ‘Method’ section of the revised manuscript. Kindly, refer to lines 205-209 on page 10 of the revised manuscript. We would like to politely recapitulate here that this information was already provided as an illustration in the original **Figure 3A** and its corresponding descriptive mechanistic explanation in the original result section, which has been retained in the revised manuscript as revised **Figure 4A** and in the text as “The rationale behind sorbitol protection assay has been depicted in **Fig 4A**. If WSWE disintegrates the cell wall **(indicated through the right arm in Fig 4A)**, it will be essentially generating protoplasts, for which the normal media will act as a hypotonic solution resulting in cell swelling and finally, bursting, However, if sorbitol is provided in the extracellular milieu **(left arm of Fig 4A)**, a cushioning effect ensues as soon as cells form protoplast upon losing cell wall due to WSWE treatment. This will prevent the protoplasts from swelling and bursting. Experimentally, the second scenario will be manifested as reduced fungicidal effect when compared to the first. There will be an increase in the IC_50_ value of WSWE in the presence of sorbitol.” Kindly, refer to lines 342-350 on page 16 of the revised manuscript.

Ergosterol binding assay helps to determine whether the test anti-fungal agent affects the fungal cell membrane by sequestering the membrane ergosterol (Mani-López et al., 2021). In this case, the 400 μg/ml ergosterol provided in the culture medium competitively inhibited the test article from binding to the membrane ergosterol, thereby, reducing its anti-fungal efficacy. This is manifested as increased IC_50_ value of the anti-fungal agent in the presence of ergosterol in the medium as compared to that determined in its absence. This rationale behind the ergosterol assay has been now included in the ‘Method’ section of the revised manuscript for better clarity. Kindly, refer to lines 216-221 on pages 10 and 11 of the revised manuscript. This rationale has been illustrated in the new **Figure 4B** and explained in the ‘Result’ section of the original and revised manuscript as “Like the sorbitol assay, the ergosterol binding assay also relies upon the shift in IC_50_ value to assess the effect on cell membrane **(Fig 4B)**. In this case, a fungicide, like, amphotericin B which binds the membrane-associated ergosterol creates pores in the cell membrane resulting in intra-cellular leakage and eventual cell death **(left arm of Fig 4B)**. But, if ergosterol is supplied in the extracellular milieu, through competitive binding the extracellular population of ergosterol will significantly reduce the fungicide available for binding the membrane-associated ergosterol. Consequently, this will lead to inefficient pore formation in the fungal cell membrane and manifest as reduced antifungal effect (increased IC_50_ value) of the fungicide **(right arm of Fig 4B)**.” Kindly, refer to lines 350-358 on pages 16 and 17 of the revised manuscript.

3. We apologize for the inadvertent error in the usage of MIC_50_ and MIC_80_. The terminologies have been replaced with IC_50_ and IC_80_ throughout the revised manuscript.
4. Phytocompounds present in WSWE were identified against commercially procured respective standards. The standards for withanoside IV, withanoside V, withaferin A and withanone were purchased from Natural Remedies Pvt. Ltd. (Bengaluru, India) whereas, those for withanolides A and B were procured from Sigma-Aldrich (Bengaluru, India). This information has been provided in the original manuscript and is retained in lines 157-160 on page 8 of the revised manuscript.

**References:**

Mani-López, E., Cortés-Zavaleta, O., and López-Malo, A. (2021). A review of the methods used to determine the target site or the mechanism of action of essential oils and their components against fungi. SN Appl. Sci. 3, 1–25. doi:10.1007/s42452-020-04102-1.

-----------------------------------------------------------------------------------------------------------------

Results
-Does the analysis presented match the analysis plan?
-Are the results clearly and completely presented?
-Are the figures (Tables, Images) of sufficient quality for clarity?

Reviewer #1: 1. Analysis presented was match with the analysis plan.
2. Results were clearly and completely presented.
3. Figures and graphs were sufficient and clearly defined.
**Authors’ Response:** We thank the reviewer for this encouraging feedback.

Reviewer #2: Results is clearly presented. They suggested making comparisons AmB vs. WSWE, and WSWE vs. Untreated, using a statistical test (Figure 2).
Trials are adequate, but I suggest making statistical comparisons (Figure 3).

**Authors’ Response:** We thank the reviewer for the positive feedback on our results. We have offered a comparative presentation of the effects exerted by amphotericin B and WSWE on growth kinetics of *Sporothrix globosa* in **Figure 2**. Originally, the comparisons were done with untreated and WSWE treated datasets. In the revised manuscript, as per the reviewer’s suggestion, the comparators are untreated and amphotericin B treated groups, and only WSWE treated group is compared. Accordingly, **Figure 2** has been revised to **New Figure 3**, as a **New Figure 2** has been included on determination of minimum fungicidal concentration (MFC) of WSWE in response to one of the suggestions from reviewer 4. Consequently, the numbers of the figures 3, 4 and 5 have increased by one. We have also included statistical analysis of significance for the observed shifts in inhibitory concentrations (IC_50_ and IC_80_) of WSWE in the presence of ergosterol and sorbitol as additional panels F and G of **New Figure 4** (originally **Figure 3**).

Reviewer #3: (No Response)

Reviewer #4: Results comply with these points
**Authors’ Response:** We thank the reviewer for this positive feedback.

-----------------------------------------------------------------------------------------------------------------
Conclusions
-Are the conclusions supported by the data presented?
-Are the limitations of analysis clearly described?
-Do the authors discuss how these data can be helpful to advance our understanding of the topic under study?
-Is public health relevance addressed?

Reviewer #1: 1. Yes conclusions were supported by data.
2. The author did not mention the limitation of study analysis.
3. The discussion part was weak in supporting the study findings for translational medicine.
4. The study did not address the public health importance in major concern.

**Authors’ Response:** We thank the reviewer for meticulously assessing the manuscript. As per the reviewer’s suggestions, limitation of the study and its impact on translational medicine and public health have now been included in the discussion. Kindly, refer to lines 462 to 481 on page 22 of the revised manuscript. These constructive suggestions from the reviewer has improved the overall quality of the manuscript.

Reviewer #2: The study should focus on the in vitro test. The first paragraph is out of context., I suggest reviewing.
I suggest expanding the discussion. Include articles on the antisporotrichotic effects of other plants.
Consider to describe that the antisporotrichotic effect of WSWE probably results from the combination of the phytochemical components or from one particular component as well.
They should also discuss further testing to identify the main phytochemical component of WSWE with antisporotrichotic activity.
Describe and discuss the main limitations of your study.
**Authors’ Response:** We thank the reviewer for these critical comments and we have tried to address them to the best of our ability. We do agree that an *in vitro* host pathogen interaction would have been a good addition. However, to our humble understanding, such studies on infection biology alone merit for independent publication. We do consider this suggestion seriously for our next phase of research on Sporotrichosis.

In order to avoid redundancy, we have integrated the first paragraph of the ‘Discussion’ section into ‘Introduction’ and have included relevant citations on anti-sporotrichotic effects of other plants, as per the reviewer’s suggestion. Kindly, refer to lines 89 to 144 on pages 5 to 7 of the revised manuscript.

In the revised manuscript, we have discussed the possible roles of identified phytocompounds in conferring anti-sporotrichotic effect on WSWE while reporting the same for withanone and withaferin A purified from WSWE. Kindly, refer to lines 432 to 437 on page 20 of the revised manuscript.

Reviewer’s suggestion to ‘discuss further testing to identify the main phytochemical component of WSWE with antisporotrichotic activity’ motivated us to purify withanone and withaferin A from WSWE and investigate their anti-sporotrichotic effects. We have included this data as **Figure 8** (shown below) in our revised submission of the manuscript.

The observation has been included in the revised manuscript as “Two of these phytocompounds, withanone and withaferin A, were purified from WSWE and were tested for their anti-sporotrichotic effects. The IC_50_ values for withanone and withaferin A against *S. globosa* were found to be 130 and 94 μg/ml, respectively **(Figure 8)**, indicating > 10 fold higher anti-sporotrichotic effect compared to WSWE. Even the observed IC_80_ values of withanone and withaferin A (287.08 and 160.06 μg/ml, respectively) were in micrograms as compared to milligrams in case of WSWE. This clearly indicated that withanone and withaferin A could be responsible for the observed anti-sporotrichotic effect of WSWE.” Kindly, refer to lines 400 to 407on pages 19 of the revised manuscript. HPLC chromatogram originally submitted as **Figure 5** is provided as **Figure 6** in the revised submission, with minor revisions in the graphics.

As per the reviewer’s suggestion, the limitations of the current study have been now included in the revised manuscript. Kindly, refer to lines 462 to 471 on page 22 of the revised manuscript.

Reviewer #3: (No Response)

Reviewer #4: Authors didnt describe the limitation of their study.
**Authors’ Response:** The limitations of the study is now included in the revised manuscript. Kindly, refer to lines 462 to 471 on page 22 of the revised manuscript.

-----------------------------------------------------------------------------------------------------------------
Editorial and Data Presentation Modifications?
Use  this section for editorial suggestions as well as relatively minor modifications of existing data that would enhance clarity. If the only modifications needed are minor and/or editorial, you may wish to recommend “Minor Revision” or “Accept”.

Reviewer #1: (No Response)

Reviewer #2: Major revision

Reviewer #3: (No Response)

Reviewer #4: I would suggest the authors that
1. In your introduction, tell less about sporotrichosis and more about the public health problem and above all more about the how is currently used? what type of previous studies have been done to study it? what is its range of activity? what previous studies have been published.
2.Discussion is repetitive compared to the introduction.
3. The authors didn't explain about how the data analysis was done? how many times these experiments were carried out?
4. Authors used only one strain, hence they should been using the terms MIC50, MIC90.
5. I think assays of minimal fungicidal concentration would enrich this WSWE manuscript. As well as TEM
6. Testing more strains would be useful
**Authors’ Response:** We sincerely thank the reviewer for critically evaluating our manuscript. We have tried our best to address the above-mentioned comments and suggestions below:

1. As per the reviewer’s suggestion, the ‘Introduction’ has been revised substantially to incorporate information on sporotrichosis as a public health problem and the available treatment and management options citing relevant references.
2. The ‘Discussion’ is also revised to remove any redundancy.
3. We would like to politely refer here the ‘Statistical Analysis’ sub-section of the ‘Method’ section wherein information on data analysis was already included as “Data represented as mean ± standard error of mean (SEM) using GraphPad Prism version 7.0 (GraphPad Prism 7.0, San Diego, CA, USA). Statistical significance of the observations was determined using in-built analysis option of the software. Observation was considered to be significant at p < 0.05.” Please refer to lines 277 to 281 on page 13 of the revised manuscript. Besides, specific statistical tests used have been mentioned in the corresponding figure legends.
4. We sincerely apologize for this inadvertent error and rectified it throughout the revised manuscript.
5. As per the reviewer’s suggestion we attempted to determine the minimum fungicidal concentration of WSWE against *Sporothrix globosa*. The *S. globosa* cells from broth microdilution cultures were plated on BHI agar plates to determine the MFC. Our observation reveals that at *S. globosa* cells treated with 50 mg/ml WSWE and above did not grow on non-selective plates. This data has been included in the revised manuscript as “When *S. globosa* cells treated with different concentrations of WSWE were plated on non-selective BHI agar plates, the minimum fungicidal concentration (MFC) was found to be 50 mg/ml **(Figure 2A & 2B)**. The mechanism of antibiosis can be determined from the ratio of MFC and the minimum inhibitory concentration (MIC) or the concentration of the anti-microbial agent offering visible microbial growth inhibition. So, 99 % inhibition (IC_99_) of *S. globosa* growth was precisely determined through non-linear regression, using the in-built analysis tool of the GraphPad Prism software, and was found to be 34.51 mg/ml. The ratio between MFC and IC_99_ (MIC in this case) being 1.45 (< 2), indicates that the mechanism of antibiosis of WSWE is most likely fungicidal (Olajuyigbe and Afolayan, 2012).” in lines 290 to 298 on page 14 and **New Figure 2**, as shown below:

A possible impact of this observation on potential clinical aspect has also been discussed in the revised manuscript. Kindly, refer to lines 476-481 on page 22 of the revised manuscript. Transmission Electron Microscopy (TEM), no doubt would add an edge to the study. However, due to technical limitations, we are afraid that we would not be able to include it in our current study.

6. Including more strains is definitely a very good idea, which we have seriously considered for our subsequent studies. We thank the reviewer for these insightful comments which not only helped in improving the quality of the current manuscript but also seeded some great future study plans.

**References:**

Olajuyigbe, O. O., and Afolayan, A. J. (2012). Pharmacological assessment of the medicinal potential of Acacia mearnsii de wild: Antimicrobial and toxicity activities. Int. J. Mol. Sci. 13, 4255–4267. doi:10.3390/ijms13044255.

----------------------------------------------------------------------------------------------------------------
Summary and General Comments
Use  this section to provide overall comments, discuss strengths/weaknesses of the study, novelty, significance, general execution and scholarship. You may also include additional comments for the author, including concerns about dual publication, research ethics, or publication ethics. If requesting major revision, please articulate the new experiments that are needed.

Reviewer #1: 1.The title was long and confusing, should be revised.
2. Limitation of the study should be discussed.
3. The result should be clearly correlate with future role in disease management (Translational medicine)

**Authors’ Response:** We once gain thank the reviewer for a meticulous assessment of our manuscript and for the constructive suggestions to enhance its quality. The above-mentioned comments from the reviewer have been addressed below to the best of our ability:

1. As per the reviewer’s suggestion, the title of the manuscript has been revised. The new title is “***Withania somnifera* (L.) Dunal Whole-plant Extracts Exhibited Anti-Sporotrichotic Effects by Destabilizing Peripheral Integrity of *Sporothrix globosa* Yeast Cells**”.
2. Limitations of the study are now included in the revised manuscript as “This study has offered several important leads for future studies to develop WSWE into a phytotherapeutic for treating Sporotrichosis. For example, the effect of WSWE on host-pathogen interactions, in terms of pathogen infectivity, host immune-modulation to confront infection, etc. could be explored using *in vitro* experimental models of infection. Development of the WSWE containing formulation is yet another avenue opened by this study. Moreover, in detail pre-clinical, toxicological and eventual clinical studies are warranted by the outcomes of this one. Although, all these studies fall beyond the scope of the current manuscript, nevertheless, they do give a limited feel to this chronicle. Besides, the observations reported in this manuscript provide strong rationale for a comprehensive study on the effect of WSWE on multiple clinically isolated strains of *Sporothrix* that would more closely resemble the pathogenic species complex responsible for causing Sporotrichosis.” Kindly, refer to the lines 462 to 471 on pages 22 of the revised manuscript.
3. A future perspective on the impact that this study might have on the disease management is also included in the revised manuscript. Kindly, refer to lines 473 to 481 on page 22 of the revised manuscript.

Reviewer #2: (No Response)

Reviewer #3: In this study Balkrishna et al., describes the antifungal activity of Withania somnifera whole plant extract against Sporothrix globose and its plausible use in treating Sporotricosis. Although this is a well written manuscript, the study described in this manuscript is limited to the preliminary observation on the crude extract’s antifungal activity against S. globose. There are several reports describing the antifungal activities of W. somnifera extracts. The current study only adds another fungal species to the existing list of fungal pathogens that are susceptible to W. somnifera extracts. Therefore, I am of the opinion that the manuscript in its current form is not suitable for the publication in PLOS Neglected Tropical Diseases and the manuscript need a major revision with potential attempts to identify the active antifungal component of the extract and its plausible mode of action.
**Authors’ Response:** We thank the reviewer for critically assessing our manuscript and offering insightful recommendations and suggestions to improve its overall quality.

Major Comments:
1)        One of the major weakness of the manuscript is the activities described are limited to the crude extract. The HPLC chromatogram given on the Fig 5, clearly indicates many separable peaks. The authors need to purify these compounds and test the activity of purified peaks to find the potential antifungal molecule/s.  Some of the molecules are commercially available, so authors may use commercially available compounds or the purified one. If no individual peaks show any activity against S. globose, the authors could then employ synergistic assays to validate the compounds activity in combinations.  If the active peak represents an unknown molecule, the authors are recommended to do the characterization of the same.

**Authors’ Response:** We thank the reviewer for this insightful suggestion and have tried and successfully purified withanone and withaferin A from WSWE and assessed their anti-sporotrichotic activities. We have observed that purified withanone and withaferin A have higher anti-fungal potencies, that showed that these phytocompounds are responsible for the observed anti-sporotrichotic property of WSWE. We have included this observation in the revised manuscript as new **Figure 8**.

Synergistic study as recommended by the reviewer is indeed as insightful suggestion and would be of even more relevance if conducted with known anti-fungal agents, against which pathogenic fungi have already developed resistance. As suggested by the reviewer, all other marker phytocompounds of WSWE can be isolated and characterized for their anti-fungal efficacies against *Sporothrix globosa*. However, to our humble understanding that would become an independent study with its own merits, deserving consideration as a separate manuscript, altogether. Therefore, we have kept this suggestion by the reviewer in perspective for our upcoming study.

The growing recognition of medicinal herbs, particularly, *Withania somnifera* and its isolated phytocompounds, as potential anti-fungal agents will certainly have far-fetched clinical impacts in the long run. Since, this paradigm shift is in its infancy, therefore, several exploratory studies are already reported (Javaid and Akhtar, 2015; Nefzi and Ben Abdallah, 2016; Khanchandani et al., 2019; Seepe et al., 2021). We would like to politely iterate here that our study stands out from other reported ones in many aspects. To begin with, this is the first report wherein the whole plant extract of *W. somnifera* has been explored for its biological functionality. Secondly, this study has shown that the *Sporothrix globosa* isolate used is recalcitrant to one of the widely used anti-fungal agent, Itraconazole, although, it retains its susceptibility to Amphotericin B. Furthermore, we have explored the mode-of-action of WSWE to gain mechanistic insight. Besides, studies reported so far have explored other plants for their anti-sporotrichotic activity, and *W. somnifera* was not in that list (Waller et al., 2016). Therefore, taken together, this study although preliminary, but is quite comprehensive in its own accord, offering, as expected, several important leads for future studies towards developing WSWE into an anti-fungal therapeutic.

**References:**

Javaid, A., and Akhtar, R. (2015). Antifungal activity of methanolic root extract of Withania sommnifera against Fusarium oxysporum f. sp. cepae. African J. Tradit. Complement. Altern. Med. 12, 22–27. doi:10.4314/ajtcam.v12i5.4.

Khanchandani, N., Shah, P., Kalwani, T., Ardeshna, A., and Dharajiya, D. (2019). Antibacterial and Antifungal Activity of Ashwagandha (Withania somnifera L.): A review. J. Drug Deliv. Ther. 9, 154–161. doi:10.22270/jddt.v9i5-s.3573.

Nefzi, A., and Ben Abdallah, R. A. (2016). Antifungal activity of aqueous and organic extracts from Withania somnifera L. against Fusarium oxysporum f. sp. radicis-lycopersici. J. Microb. Biochem. Technol. 8, 144–150. doi:10.4172/1948-5948.1000277.

Seepe, H. A., Ramakadi, T. G., Lebepe, C. M., Amoo, S. O., and Nxumalo, W. (2021). Antifungal Activity of Isolated Compounds from the Leaves of Combretum erythrophyllum (Burch.) Sond. and Withania somnifera (L.) Dunal against Fusarium Pathogens. Molecules 26, 1–21. doi:10.3390/molecules26164732.

Waller, S. B., Madrid, I. M., Faria, R. O. De, Cleff, M. B., Roberto, J., Mello, B. De, et al. (2016). Anti- Sporothrix spp . activity of medicinal plants. Brazilian J. Pharm. Sci. 52, 221–238. doi:10.1590/S1984-82502016000200001.

  
2)         On page 13 lines 273-281 and Fig 3 D: The authors used Sorbitol and ergosterol to assess the potential mode of action of WSWE. The results shown in the Figure and text given indicates that there is only a 2-3 folds difference in the MFC50 in the presence of these molecules. The authors are suggested to do statistical analysis of their data given on Fig 3D to validate whether that difference is significant. Also, data given on the Fig3D further indicate that at MFC80 the difference will be meager, unlike AMB data, where difference between +/- Ergosterol is significantly different. I would also suggest the authors to include the growth curves of the S. globose with and without ergosterol and sorbitol as supplementary document, so that readers are aware that these compounds doesn’t affect the overall growth curve.

**Authors’ Response:** We thank the reviewer for this insightful comment. As per the suggestion, we have conducted statistical analysis to determine the significance of the observed differences in IC_50_ values of WSWE in the presence and absence of sorbitol/ergosterol. This information is now included in the revised manuscript and in new **Figure 4F**. Kindly, refer to lines 360 and 370 on page 15 of the revised manuscript. We also looked into the reviewer’s observation…

“Also, data given on the Fig3D further indicate that at MFC80 the difference will be meagre, unlike AMB data, where difference between +/- Ergosterol is significantly different.”

…and analysed the changes in IC_80_ values of WSWE in the presence of ergosterol and sorbitol. We noted that corresponding IC_80_ values of WSWE also showed fold increases similar to the ones pertaining to IC_50_ as indicated in new **Figure 4E** and graphically represented in **Figure 4G**. This new analysis in now included in the revised manuscript. to lines 360 and 370 on page 15 of the revised manuscript. Furthermore, as per the reviewer’s suggestion, we also assessed the effect of ergosterol or sorbitol alone on *Sporothrix globosa* cells during ergosterol binding and sorbitol protections assays, respectively. We observed that the growth of the cells was not affected by ergosterol or sorbitol alone, as shown in new **Figure 4C** and captured in line 358 on page 17 of the revised manuscript.

Moreover, delving further into this mode-of-action, as a proof of the hypothesis, total ergosterol content of the WSWE treated and untreated cells was determined and was found to decrease in WSWE cells. This observation is now included in the revised manuscript as a new **Figure 5**, as shown below, …and mentioned in the text as “Furthermore, in order to verify these observations, effect on overall content of ergosterol in *S. globosa* cells treated with AMB or different concentrations of WSWE was analysed through HPLC **(Fig 5A)**. Just like, AMB treatment reduced the overall content of ergosterol in *S. globosa* cells, so did, WSWE, in a dose-dependent manner **(Fig 5B)**.” and captured in lines 370 to 373 on page 17 of the revised manuscript.

Minor comments:
1)        Page 12 lines 247-249: “In S. globosa, like other fungi, amphotercin B is known to bind the membrane-associated ergosterol creating pores leading to leakage of intracellular contents and eventual cell death [11,17]. This indicated that WSWE could be affecting cell membrane”. The text or data preceding this statement doesn’t indicate WSWE affect the membrane integrity. Being more active against metabolically active cells doesn’t imply that mode of action of AMB and WEWE are similar. In fact, many known antimicrobial agents show potent activity towards metabolically active cells.

**Authors’ Response:** We sincerely apologize for this lack of clarity. We have now revised the text as “The *S. globosa* strain being studied has shown resistance to itraconazole, that functions as an antifungal agent by targeting ergosterol biosynthesis [12], whereas, it was susceptible to amphotericin B treatment. In *S. globosa*, like other fungi, amphotericin B is known to bind the membrane-associated ergosterol creating pores leading to leakage of intracellular contents and eventual cell death [12,18]. The fact that WSWE affected *S. globosa* cells, in a manner similar to amphotericin B, it would be worthwhile to check whether both shares a common mode-of- action as anti-fungal agents.” We hope that rephrasing the content has infused the required contextual clarity that the subsequent experiments, to assess effects of WSWE on cell permeability, were primarily based on the premise that WSWE and amphotericin B, might share a similar mode of antifungal activity. We thank the reviewer for assessing our manuscript meticulously, as a result of which we got this and many more similar suggestions to improve the lucidity of the manuscript.

2)        Page 17 lines 366-373: It is not clear what the authors are referring to. None of the figures or texts given in the manuscript indicates the given values. Further, the authors need to use caution while claiming a multi target mechanism for WESE extract, as there is no solid evidence for any target.

**Authors’ Response:** Our apologies for this inadvertent confusion. The reviewer refers to the following segment in the discussion section of the manuscript:

“Positive Hill co-efficients of 2.77 ± 0.76 and 1.35 ± 0.26 for amphotericin B and WSWE, respectively, indicate positive cooperativity of ligand binding in both the cases. The slightly steeper Hill slope of amphotericin B compared to WSWE (2.77 *versus* 1.35) suggested more cooperativity in case of ligand binding of amphotericin B which might eventually translate into improved drug response. In hindsight, higher cooperativity for ligand binding suggests increased target specificity with increased chances of drug recalcitrance. Lower target specificity as shown by less steep Hill slope/ lower Hill co-efficient in case of WSWE suggests broader target specificity and lesser chances of evolving drug resistance [29].”

The figures mentioned here are the GraphPad Software generated Hill co-efficient values for non-linear regression analyses conducted to obtain the dose response curves for anti-fungal effects of amphotericin B and WSWE against *Sporothrix globosa*. In order to eliminate this confusion and as per the reviewer’s suggestion to exercise caution regarding ‘multi-target mechanism’, the text has been revised as follows:

“The GraphPad Prism software-mediated non-linear regression analyses for obtaining dose response curves for the anti-sporotrichotic effects of amphotericin B and WSWE generated Hill co-efficient values. Positive Hill co-efficients of 2.77 ± 0.76 and 1.35 ± 0.26 for amphotericin B and WSWE, respectively, indicate positive cooperativity of ligand binding in both the cases. The slightly steeper Hill slope of amphotericin B compared to WSWE (2.77 *versus* 1.35) suggested more cooperativity in case of ligand binding of amphotericin B which might eventually translate into improved drug response. In hindsight, higher cooperativity for ligand binding suggests increased target specificity with increased chances of drug recalcitrance. Lower target specificity as shown by less steep Hill slope/ lower Hill co-efficient in case of WSWE might suggest broader target specificity and thereby, lesser chances of evolving drug resistance (Talevi, 2015).” Kindly, refer to lines 450 to 459 on pages 21 of the revised manuscript.

**References:**

Talevi, A. (2015). Multi-target pharmacology: Possibilities and limitations of the “skeleton key approach” from a medicinal chemist perspective. Front. Pharmacol. 6, 1–7. doi:10.3389/fphar.2015.00205.

Reviewer #4: This is as study evaluating the anti-fungal effect of whole extract of W. somnifera on a single S. globosa strain.
 
Questions in Methods sections: 1. Is fungal culture medium the same than BHI? or are you referring to a different medium? please specify and comment the composition of the medium. 2) The rational behind ergosterol and sorbitol assays is not clear. Please specify. 3) I think the terms MIC50 and MIC80 are wrongly used here. 4) what were the standards used for the HPLC analysis and how is that you choose them? This section is not clear. how you identified withaferin, withanoline etc?

**Authors’ Response:** We sincerely thank the reviewer for going through our manuscript with a fine comb and offering insightful suggestions to improve its overall quality. To best of our ability, we have addressed the above-mentioned comments below:

1. Brain Heart Infusion (BHI) agar or broth, as per experimental requirement, has been used in this study.
2. Sorbitol protection assay relies on the isotonic protection provided by 0.8 M sorbitol in the medium to the cells whose cell walls have been damaged by the test anti-fungal agent. Due to this protection, in the presence of sorbitol, the efficacy of the anti-fungal agent is apparently reduced as evident from increased IC_50_ value in comparison to that obtained for the same fungus in the absence of sorbitol. Ergosterol binding assay helps to determine whether the test anti-fungal agent affects the fungal cell membrane by sequestering the membrane ergosterol. In this case, the 400 μg/ml ergosterol provided in the culture medium competitively inhibited the test article from binding to the membrane ergosterol, thereby, reducing its anti-fungal efficacy. This is manifested as increased IC_50_ value of the anti-fungal agent in the presence of ergosterol in the medium as compared to that determined in its absence.
3. We apologize for the inadvertent error in the usage of MIC_50_ and MIC_80_. The terminologies have been replaced with IC_50_ and IC_80_ throughout the revised manuscript.
4. Phytocompounds present in WSWE were identified against commercially procured respective standards. The standards for withanoside IV, withanoside V, withaferin A and withanone were purchased from Natural Remedies Pvt. Ltd. (Bengaluru, India) whereas, those for withanolides A and B were procured from Sigma-Aldrich (Bengaluru, India). This information has been provided in the original manuscript and is retained in lines 157-160 on page 8 of the revised manuscript.

I would suggest the authors that
1. In your introduction, tell less about sporotrichosis and more about the public health problem and above all more about the how is currently used? what type of previous studies have been done to study it? what is its range of activity? what previous studies have been published.

**Authors’ Response:** Introduction in the revised manuscript has been modified as per the reviewer’s suggestion.

2.Discussion is repetitive compared to the introduction.

**Authors’ Response:** Discussion has been revised as per the reviewer’s suggestion.
3. The authors didn't explain about how the data analysis was done? how many times these experiments were carried out?

**Authors’ Response:** A detailed description of data analysis and information on conduction of experiments in replicates is provided under ‘Statistical Analysis’ of the Methods section.
4. Authors used only one strain, hence they should been using the terms MIC50, MIC90.

**Authors’ Response:** We have replaced MIC with IC throughout the revised manuscript as per the reviewer’s suggestion.

5. I think assays of minimal fungicidal concentration would enrich this WSWE manuscript. As well as TEM before and after the efect of WSWE

**Authors’ Response:** As per the reviewer’s suggestion we attempted to determine the minimum fungicidal concentration of WSWE against *Sporothrix globosa*. Our observation reveals that WSWE is fungicidal in nature. This additional information is now included in the revised manuscript text in 290 to 298 on page 14 as well as in revised **New Figure 2** as representative microbiological plate images.

We agree with the reviewer that Transmission Electron Microscopy (TEM) would add quality to the study. Unfortunately, due to technical limitations, it would not be possible to include it in our current study. But we take this insightful technical advice into consideration in general for our other studies.

6. Testing more strains would be useful

**Authors’ Response:** We thank the reviewer for this suggestion which seeded some great future study plans in our minds. This review comment made us realize that inclusion of multiple clinically isolated strains of *S. globosa* should be an integral part of the next study plan.
7. Determination of which component out of the ones found by HPLC is the one with the real anti-fungal capacity.

**Authors’ Response:** As per the reviewer’s suggestion, we purified withanone and withaferin A from WSWE and evaluated their anti-fungal activities against *Sporothrix globosa*. This additional data is now included in the revised manuscript as new **Figure 8**.
